# Antidiabetic Activities of *Abutilon indicum* (L.) Sweet Are Mediated by Enhancement of Adipocyte Differentiation and Activation of the GLUT1 Promoter

**DOI:** 10.1093/ecam/neq004

**Published:** 2011-02-17

**Authors:** Chutwadee Krisanapun, Seong-Ho Lee, Penchom Peungvicha, Rungravi Temsiririrkkul, Seung Joon Baek

**Affiliations:** ^1^Department of Pathobiology, College of Veterinary Medicine, University of Tennessee, 2407 River Drive, Knoxville, TN 37996, USA; ^2^Department of Physiology, Faculty of Pharmacy, Mahidol University, Bangkok, Thailand; ^3^Department of Pharmaceutical Botany, Faculty of Pharmacy, Mahidol University, Bangkok, Thailand

## Abstract

*Abutilon indicum* (L.) Sweet is an Asian phytomedicine traditionally used to treat several disorders, including diabetes mellitus. However, molecular mechanisms supporting the antidiabetic effect of *A. indicum* L. remain unknown. The aim of this study was to evaluate whether extract of *A. indicum* L. improves insulin sensitivity. First, we observed the antidiabetic activity of aqueous extract of the entire plant (leaves, twigs and roots) of *A. indicum* L. on postprandial plasma glucose in diabetic rats. The subsequent experiments revealed that butanol fractions of the extract bind to PPAR**γ** and activate 3T3-L1 differentiation. To measure glucose uptake enhanced by insulin-like activity, we used rat diaphragm incubated with various concentrations of the crude extract and found that the extract enhances glucose consumption in the incubated solution. Our data also indicate that the crude extract and the fractions (water and butanol) did not affect the activity of kinases involved in Akt and GSK-3**β** pathways; however, the reporter assay showed that the crude extract could activate glucose transporter 1 (GLUT1) promoter activity. These results suggest that the extract from *A. indicum* L. may be beneficial for reducing insulin resistance through its potency in regulating adipocyte differentiation through PPAR**γ** agonist activity, and increasing glucose utilization via GLUT1.

## 1. Introduction

Diabetes mellitus (DM) is a group of metabolic diseases characterized by hyperglycemia resulting from defects in insulin secretion, insulin action, or both; the incidence of diabetes is increasing worldwide. Diabetes or obesity causes substantial morbidity, mortality and long-term complications, and remains an important risk factor for cardiovascular disease [1]. There exists a genetic predisposition to diabetes; however, environmental factors such as a sedentary lifestyle and obesity are the most prominent risk factors [2]. Insulin resistance is a key feature in type 2 DM as well as in obesity and plays an important pathophysiological role [3]. Insulin resistance is characterized by a diminished reaction of insulin-sensitive tissues and a marked decrease in glucose metabolism in response to insulin. In fact, impairment of insulin action has also been observed in syndromes including inflammatory and chronic infectious diseases in addition to type 2 DM [4]. Recent data suggest that activation of PPAR*γ* by using plant extracts improves lipid metabolism and mitigates insulin resistance [5]. Throughout the world, many traditional plant treatments for diabetes are employed that are frequently considered to be less toxic and have fewer side effects than synthetic drug treatments [1, 6]. According to a medicinal survey report in Thailand, traditional medicines or herbs are used 31% of the time in the treatment of diabetic patients [7]. However, traditional antidiabetic plants possess less adequate scientific or medicinal data support in spite of the fact that the World Health Organization still recommends further evaluation of their accepted use [8, 9].

The native Southeast Asian plant *A. indicum* (L.) Sweet is an erect, branched shrub of 0.5–1 m height, having the Thai name Krob-Fun-Si, Fun-Si or Ma-Kong-Khaao. *A. indicum* L. is in the family Malvaceae, is easily identified by its cog-like fruits, and is found abundantly in wastelands. This plant has a long history of being used medicinally as an antidiabetic remedy, and phytochemical screening of the plant revealed that it contained alkaloids, flavonoids, tannins, saponins and glycosides [10, 11]. This medicinal plant plays an important role in folk medicine; in Thailand, it has been used as a blood tonic, carminative, antipyretic, anti-cough, diuretic, anti-inflammatory, laxative and antidiabetic [12], whereas in India and China, it has been used for urinary disease, gonorrhea, jaundice, rheumatism, high fever, mumps, pulmonary tuberculosis, bronchitis, lack of urination and some nervous and ear problems [13, 14]. According to many scientific reports, the leaf extracts possess hypoglycemic, hepatoprotective, antibacterial and larvicidal properties [10, 13, 15, 16]. Analgesic principles from this plant were also isolated [17], and its polyherbal formulations have been reported as being effective in treating diabetes and hyperlipidemia, and effective as free radical scavengers [18–20].

With respect to its traditional use, *A. indicum* L. has been known to contain an active ingredient against diabetes and is believed to reduce some symptoms of diabetic complications. In this study, we investigated whether the extracts of *A. indicum* L. could reduce postprandial plasma glucose levels in an animal model of diabetes, since postprandial hyperglycemia is one of the common complications seen in diabetic patients [21]. We found that *A. indicum* L. ameliorates insulin resistance via PPAR*γ* activation and enhances glucose uptake.

## 2. Methods

### 2.1. Plant Material

The leaves, twigs and roots of *A. indicum* (L.) Sweet were collected from Mahidol University, Salaya Campus, Nakorn Pathom Province, Thailand, during May 2004, and were authenticated by Rungravi Temsiririrkkul, Department of Pharmaceutical Botany, Faculty of Pharmacy, Mahidol University. A voucher specimen (PBM 04765) was deposited at the Pharmaceutical Botany Mahidol University herbarium. All fresh parts were dried at 50°C in a hot air oven and then pulverized by an electric blender. Crude extract was obtained by boiling 1 kg of plant powder (leaves 261.2 g, twigs 559.7 g and root 179.1 g) in 10 l of distilled water for 10 min. The boiling process was repeated twice, and the combined extract was filtered across cotton wool and gauze. The filtrate was lyophilized in a freeze dry system resulting in a dark brown powder of 11.44% w/w. Lyophilized extract was preserved in an airtight bottle at room temperature and protected from light until use. The crude extract (10 g) was subsequently partitioned between butan-1-ol (3 × 0.2 l) and distilled water (0.2 l). The butanol fraction was concentrated under reduced pressure in a rotary evaporator and lyophilized to give a total semi-solid residue (19% of dried crude extract). The resulting water fraction was freeze-dried, followed by generating brown powder (65.4% of dried crude extract).

### 2.2. Animals

Male Wistar rats, weighing 140–180 g, were purchased from the National Laboratory Animal Center, Mahidol University, Salaya, Nakorn Pathom Province, Thailand. They were housed in a temperature-controlled room (22–25°C) with a 12 h light-dark cycle for at least 1 week to acclimatization. The animals were fed with free access to a commercial pellet diet (C.P. Thailand) and water *ad libitum*. The animal protocol was approved by the Animal Ethics Committee, Faculty of Pharmacy, Mahidol University (No. 5/2548).

### 2.3. Preparation of Diabetic Rats

Diabetes was induced in the 16 h-fasted male Wistar rats with a single intraperitoneal injection of 75 mg kg^−1^ streptozotocin (STZ), freshly dissolved in isotonic saline. Five days after injection, diabetic rats were identified by measuring fasting plasma glucose (FPG) levels using PGO enzyme (Sigma). Rats with FPG levels over 200 mg dl^−1^ were used in the experiment [22, 23].

### 2.4. Antidiabetic Activity of *A. indicum* Extract on Postprandial Plasma Glucose

The STZ-induced diabetic rats were randomly allocated into four groups (6–9 rats per group) *A. indicum* crude extract and glibenclamide were dissolved in distilled water and administered orally once a day for 2 weeks as follows: Group 1: diabetic rats treated orally with distilled water (control), Group 2: diabetic rats treated orally with glibenclamide 5 mg kg^−1^ body weight (positive control), Groups 3 and 4: diabetic rats treated orally with crude extract 0.25 or 0.5 g kg^−1^ body weight. Blood samples (60 *μ*l of each) were collected from the tail vein using Hematocrit-capillary tube, after 2 h fasting on day 0 (1 day before oral administration), day 7 and day 14 for plasma glucose determination. The animals were weighed on day 0, 4, 7, 11 and 14 for corrected doses of medication.

### 2.5. Cell Cultures

HCT-116, HepG2, C2C12, 3T3-L1 and L6 cells were purchased from American Type Culture Collection (Manassas, VA, USA). HCT-116 cells were maintained in McCoy's 5A medium supplemented with 10% FBS; all others were maintained in DMEM supplemented with 10% FBS. Cells were grown at 37°C in a humidified, 5% CO_2_ environment. For adipocyte differentiation, differentiation medium containing 0.5 mM isobutyl methylxanthine, 1 *μ*M dexamethasone and 1 *μ*g ml^−1^ insulin was added to the culture. The differentiation media was changed every 2 days until day 4. Thereafter, DMEM containing 10% FBS and insulin was subsequently replaced every 2 days until day 8. C2C12 and L6 myoblasts were differentiated with DMEM containing 2% horse serum for 6 and 2 days, respectively.

### 2.6. Binding Activity of *A. indicum* Extract to PPAR*α* and PPAR*γ*

HCT-116 cells were plated in 12-well plates at a concentration of 2 × 10^5^ cells/well. After growth for 18 h, transient transfections were performed using lipofectamine (Invitrogen, Carlsbad, CA, USA) according to the manufacturer's instructions. MH 100×4-TK-LUC plasmid, pCMX-Gal-mPPAR*α*-LBD, and pCMX-Gal-mPPAR*γ*-LBD (0.25 *μ*g each) were co-transfected with pRL-null vector (0.05 *μ*g) into the cells. The transfected cells were cultured in the absence or presence of various concentrations of crude extract, water or butanol fraction for 24 h. Cells were harvested in 1× luciferase lysis buffer, and luciferase activity was normalized to the pRL null luciferase activity using a dual luciferase assay kit (Promega, Madison, WI, USA). Assays were performed in triplicate for each experiment group as previously described [24].

### 2.7. Analysis of mRNA Expression during Adipocyte Differentiation

Total RNA was extracted from 3T3-L1 cells at day 0 (before differentiation), 2, 4, 6 and 8 after differentiation using the PerfectPure RNA purification kit according to the manufacturer's instructions (5Prime, Gaithersburg, MD, USA). Reverse transcription (RT) was performed on 1 *μ*g of RNA samples using an iScript cDNA synthesis kit in a final volume of 20 *μ*l, according to the manufacturer's instructions (Bio Rad, Hercules, CA, USA). Specific oligonucleotide primers were designed using the Primer3 program (http://frodo.wi.mit.edu/cgi-bin/primer3/primer3_www.cgi). Quantitative changes in mRNA expression were assessed with a semi-quantitative RT-PCR, using specific primers (Table 1). The PCR products were separated electrophoretically in a 2% agarose gel and stained with ethidium bromide. 

### 2.8. Glucose Consumption Assay

Adult male Wistar rats weighing 180–200 g were used in this experiment. Animals were fasted for 36 h and sacrificed by inhalation of diethyl ether. The thorax was opened, and the diaphragm was removed. The diaphragm was immediately placed in oxygenated nutrient solution (125 ml of aerated 1.3% NaHCO_3_ was added to 750 ml of 0.16 M NaCl, 5.4 mM KCl, 2.7 mM CaCl_2_, 4.2 mM NaHCO_3_, 1.4 mM MgSO_4_ *·*7H_2_O, and 1.5 mM KH_2_PO_4_). The diaphragm was cleaned of fat and connective tissue and cut into 6 pieces before being suspended in a nutrient solution organ bath containing 300 mg dl^−1^ of glucose and bubbled with carbogen at 37°C [25]. Then, the crude extracts or insulin (used as the positive control) was added as follows: Group 1: incubated in only nutrient solution (control group), Group 2: crude extract at a concentration of 0.04 mg dl^−1^, Group 3: crude extract at a concentration of 0.2 mg dl^−1^, Group 4: crude extract at a concentration of 1.0 mg dl^−1^, Group 5: crude extract at a concentration of 5.0 mg dl^−1^, and Group 6: insulin 1 unit ml^−1^ (positive control). After 90 min of incubation, the concentration of remaining glucose in the solution was measured, and each piece of diaphragm was dried. The results were expressed as glucose consumption per 10 mg dry diaphragm.

### 2.9. GLUT1 Luciferase Reporter Assay

L6 myocytes in 48-well plates were co-transfected using FuGENE6 (Roche, Indianapolis, IN, USA) with 0.25 *μ*g GLUT1 promoter (generously provided by Dr Birnbaum at the University of Pennsylvania, Philadelphia, PA, USA) and 0.025 *μ*g of pRL-SV40 plasmid. After 30 h of transfection, cells were exposed to various concentrations of crude extract in HEPES-buffered saline, pH 7.4, containing 2% horse serum with 5 mM glucose for 16 h. The cells were washed twice with PBS and then assayed for luciferase activity as shown above.

### 2.10. Statistical Analysis

The data were presented as the mean ± standard error of the mean (SEM). Statistical analysis was performed using the Student's *t*-test or analysis of variance (ANOVA) followed by the least significant differences (LSD) test. The significance of differences was set at *P*-value < .05.

## 3. Results

### 3.1. Postprandial Plasma Glucose in STZ-Induced Diabetic Rats

**Figure 1:**
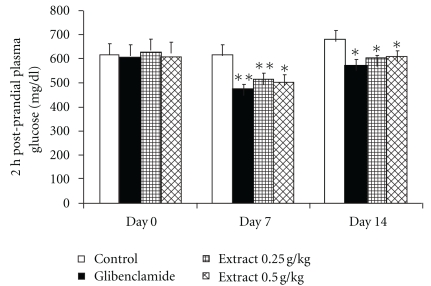
The effect of crude extract of *A. indicum* on 2 h postprandial plasma glucose in STZ-induced diabetic rats. The results show the mean ± SEM, (*n* = 6–9). Asterisks represents statistical significance (*P* < .05) compared with control at each day. Double asterisk represent statistical significance (*P* < .05) both compared with control at each day and day 0 within the group.

As shown in Figure 1, administration of *A. indicum* crude extract significantly lowered (*P* < .05) 2 h postprandial plasma glucose concentrations of treated groups in comparison to those of control STZ-diabetic rats at the corresponding time. A similar significant decrease was also observed in rats treated with glibenclamide. However, prior to treatment administration (day 0), basal plasma glucose concentration did not significantly differ between groups.

### 3.2. Binding Activity to Peroxisome Proliferator-Activated Receptors

**Figure 2:**
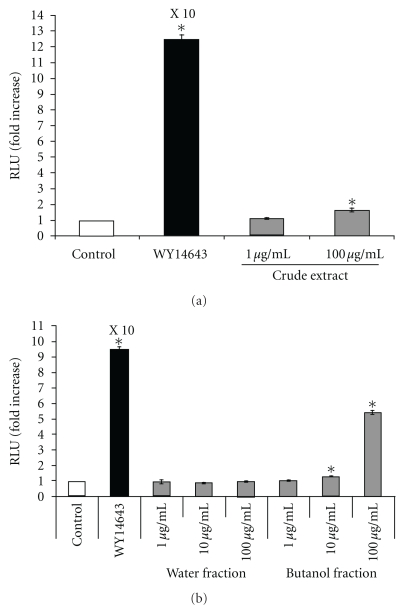
(a) The effect of *A. indicum* crude extract on luciferase activity of PPAR*α*. After the cells were transiently transfected with four copies of a Gal4 binding site (MH100×4-TK-LUC) and chimeric receptors pCMX-Gal-mPPAR*α*-LBD, the cells were treated with vehicle (DMSO, control), WY14643 (positive control) or different concentrations of crude extract for 24 h and luciferase activity was measured. The data are presented as relative luciferase activity (firefly luciferase signal/renilla luciferase signal). The results show the mean ± SEM of three independent transfections. *P < .01 versus vehicle-treated cells. (b) The effect of water and butanol fractions on luciferase activity of PPAR*α*. The data are presented as relative luciferase activity (firefly luciferase signal/renilla luciferase signal). The results show the mean ± SEM of three independent transfections. *P < .01 versus vehicle-treated cells.

**Figure 3:**
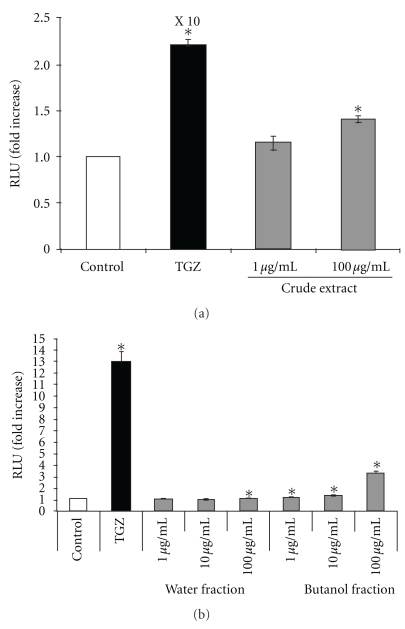
(a) The effect of *A. indicum* crude extract on luciferase activity of PPAR*γ*. After the cells were transiently transfected with four copies of a Gal4 binding site (MH100×4-TK-LUC) and chimeric receptors pCMX-Gal-mPPAR*γ*-LBD, they were treated with vehicle (DMSO, control), TGZ (positive control) or different concentrations of crude extract for 24 h and luciferase activity was measured. The data are presented as relative luciferase activity (firefly luciferase signal/renilla luciferase signal). The results show the mean ± SEM of three independent transfections. *P < .01 versus vehicle-treated cells. (b) The effect of water and butanol fractions on luciferase activity of PPAR*γ*. The data are presented as relative luciferase activity (firefly luciferase signal/renilla luciferase signal). The results show the mean ± SEM of three independent transfections. *P < .01 versus vehicle-treated cells.

To examine whether *A. indicum* extracts activate peroxisome proliferator-activated receptor (PPAR) responsiveness, HCT-116 cells were transfected with four copies of a Gal4 binding site (MH100x4-TK-LUC) and chimeric receptors (pCMX-Gal-mPPAR*α*-LBD or pCMX-Gal-mPPAR*γ*-LBD). In this system, when a compound binds to the ligand binding domain (LBD) of the PPAR*α* or PPAR*γ* chimeric receptor, the DNA binding domain of the yeast Gal4 binds to the co-transfected Gal4 binding site and initiates transcription of the firefly luciferase (LUC). After cells were treated with crude extract for 24 h, luciferase activities were measured to assess the transactivation for each PPAR receptor. As shown in Figures 2(a) and 3(a), crude extract 100 *μ*g ml^−1^ showed 1.6- and 1.4-fold increases of transactivation to PPAR*α* and PPAR*γ*, respectively, indicating that crude extract contains compounds that bind to PPAR*α* and PPAR*γ*.

The activation of PPAR*α* and PPAR*γ* using water and butanol fractions was also investigated. The results revealed that the water fraction has a minimal effect on PPAR*α* and PPAR*γ* activation, whereas butanol fractions showed a dose-dependent increase of PPAR*α* and PPAR*γ* activation (Figures 2(b) and 3(b)). The concentration of butanol fraction (100 *μ*g ml^−1^) showed 5.5- and 3.2-fold increases of transactivation to PPAR*α* and PPAR*γ*, respectively. These results suggest that the butanol fraction, but not the water fraction, contains a substance that activates PPAR*α* and PPAR*γ*.

### 3.3. mRNA Expression during Preadipocyte Differentiation

**Figure 4:**
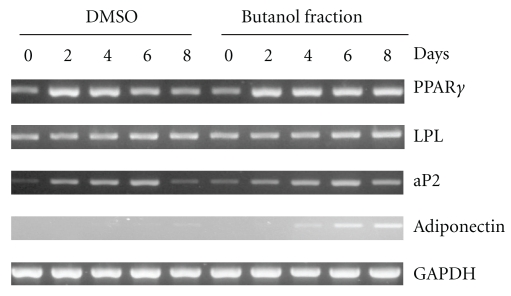
The effect of butanol fraction of *A. indicum* to induce mRNA expression of the PPAR*γ* target gene. 3T3-L1 preadipocyte cells were differentiated. Twenty-four hours before and during differentiation, cells were treated with vehicle control (DMSO) or 100 *μ*g ml^−1^ of butanol fraction. mRNA expression of PPAR*γ*, LPL, aP2, adiponectin and glyceraldehyde 3-phosphate dehydrogenase (GAPDH) were measured at Days 0, 2, 4, 6 and 8 of differentiation.

Since butanol fraction binds to PPAR*γ* and transactivates PPAR*γ* activity, gene expression profiles were observed to elucidate whether the butanol fraction actually induces adipocyte differentiation through PPAR*γ* activation. Twenty-four hours before and during differentiation, butanol fraction at 100 *μ*g ml^−1^ was added to the medium for observation of its effects on gene expression during 3T3-L1 adipocyte differentiation. Dimethyl sulfoxide (DMSO) was used as vehicle control. As shown in Figure 4, cells treated with the butanol fraction increased the expression of PPAR*γ*, aP2, and adiponectin at day 8, consistent with the previous data indicating that butanol fraction enhances PPAR*γ* activity (Figure 3(b)). However, there was no change in mRNA level of lipoprotein lipase (LPL) throughout the differentiation.

### 3.4. Glucose Consumption

**Figure 5:**
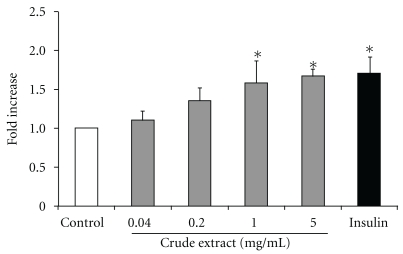
Effect of *A. indicum* crude extract on muscle glucose consumption. Diaphragms were isolated from fasted Wistar rats and incubated with 300 mg dl^−1^ of glucose in the absence or presence of crude extract for 90 min. The glucose consumption was expressed as disappeared glucose into diaphragm per 10 mg dry diaphragm. Data are presented as means ± SEM of eight replicates. Statistical analysis was performed using ANOVA followed by LSD. *P < .05 compared with the untreated group.

Crude extract of *A. indicum* was tested for insulin-like effect using diaphragm of rat. Pieces of diaphragms were incubated with various concentrations of the crude extract for 1.5 h. As shown in Figure 5, the extract enhanced glucose uptake in a concentration-dependent manner. The extract between concentrations of 1–5 mg ml^−1^ significantly increased glucose uptake and showed an almost maximal effect on glucose uptake at the concentration of 1 mg ml^−1^. Insulin was used as a positive control and also showed an *∼*1.7-fold induction over the untreated group.

### 3.5. Protein Expression in Insulin Signaling Pathway and GLUT1 Promoter Activation

**Figure 6:**
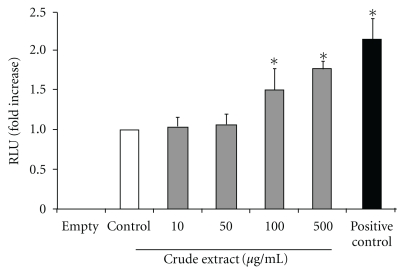
The effect of *A. indicum* crude extract on the transcription level of *Glut1*. L6 myocytes were transfected with GLUT1 promoter and pRL-SV40 plasmid. Then, cells were exposed to indicate concentrations of crude extract for 16 h as described in the ‘Methods' section. The luciferase activity was expressed as a ratio of firefly to renilla luciferase activity. Transfections and assays were performed in triplicate for each experiment group. Data showed mean ± SEM of fold increase of three replicates against control group. *P < .05 compared with untreated sample group. The pGL3-Basic vector (empty) was used as a negative control, and pGL3-control vector was used for a positive control.

Since protein kinases B (Akt) and glycogen synthase kinase 3*β* (GSK3*β*) are major kinases of the insulin signaling pathway [26, 27], the effects of crude extract and butanol and water fractions on phosphorylation of Akt and GSK3*β* in C2C12 myotubes and 3T3-L1 adipocytes were examined. However, no changes were detected in Akt phosphorylation or GSK3*β* phosphorylation, although insulin activated these two biochemical markers (data not shown), suggesting that extracts and fractions do not affect the Akt/GSK3*β* pathway. To determine the effect of crude extract on the transcriptional activity of *Glut1*, a pGL3-GLUT1 luciferase construct that contains –2100 bp of the rat Glut1 promoter region was transfected into L6 cells. After transfection, the cells were treated with various concentrations of crude extract for 16 h, and luciferase activity was measured. As shown in Figure 6, the cells treated with the extract increased relative luciferase unit (RLU) in a dose-response manner. A significant fold increase against untreated cells was found in the cells treated with 100 and 500 *μ*g ml^−1^ of crude extract.

## 4. Discussion

Much research effort is currently directed toward development of adipogenesis and lipogenesis studies, and a number of antidiabetic compounds have been developed and tested for their effects. For example, thiazolidinediones (TZDs), synthetic PPAR*γ* ligands, are a novel class of antidiabetic drugs for patients with type 2 diabetes, and two of these, rosiglitazone and pioglitazone, are currently available for clinical use [28]. Originally, TZDs were identified based on their antihyperglycemic activity, but they are also able to improve other abnormalities associated with type 2 diabetes, such as hyperlipidemia, atherosclerosis, hypertension, chronic inflammation and fibrinolytic state. It is well established that PPAR*γ* agonists such as TZDs promote the adipogenesis of 3T3-L1 cells, which have been used for the development of antidiabetic compounds [29–31]. Since troglitazone (TGZ) was voluntarily withdrawn from the market in March 2000 due to occurrence of severe idiosyncratic liver injury, it is necessary to establish a safer compound that shows antidiabetic activity. Many researchers have focused on the study of dietary phytochemicals found in plants. In this report, we examined whether the extract of *A. indicum* has antidiabetic activity in STZ-induced diabetic rats. The results demonstrated that administration of *A. indicum* extract at 0.25 and 0.5 g kg^−1^ resulted in significant decreases of postprandial plasma glucose, as seen in glibenclamide-treated rats. Although glibenclamide lowers plasma glucose level, mainly by stimulating insulin secretion from pancreatic *β*-cells [32], destruction of *β*-cells are observed in STZ-induced diabetes model (type 1 diabetes) [33]. Thus, extrapancreatic action such as stimulating liver and muscle fructose-2,6-bisphosphate formation may be involved in the effect of both glibenclamide and extracts used in this study rather than insulin secretion. In fact, it has been known that the extrapancreatic action of glibenclamide has an important role to inhibit glucose production by liver and improve carbohydrate metabolism in diabetes [34, 35], and increases glucose disposal by stabilizing GLUT1 protein at the plasma membrane of muscle [36]. Subsequently, we found that crude extract of *A. indicum* acts as a PPAR*γ* ligand. Further, the data indicated that the butanol fraction (non-polar part) may contain a compound that induces PPAR*γ* activity (Figure 3(b)). The present study also demonstrates that the butanol fraction enhances 3T3-L1 adipocyte differentiation by an increase of accumulation of differentiation and fat cell marker genes (Figure 4). The accumulation of these mRNA reflects both the rate of initiation of transcription and the half time of degradation of those transcripts.

At the molecular level, adipogenesis is driven by a complex transcriptional cascade involving the sequential activation of CCAAT/enhancer binding proteins (C/EBPs) and PPAR*γ* [37]. Loss-of-function studies have convincingly shown that PPAR*γ* is necessary as well as sufficient to promote adipogenesis and that C/EBP*α* is influential by maintaining the expression of PPAR*γ* and promoting full insulin sensitivity [38]. Our data indicate that exposing 3T3-L1 preadipocytes to the butanol fraction during adipogenesis increases PPAR*γ* mRNA, as well as the adipocyte-specific fatty acid binding protein (aP2) mRNA level; aP2 is one of the genes that characterizes the adipocyte phenotype [37, 39]. Interestingly, the mRNA level of adiponectin in the butanol fraction-treated group was higher than the control throughout the differentiation. Adiponectin is exclusively expressed by mature adipocytes and is the most abundant circulating adipokine. Thus, these data imply that the butanol fraction of *A. indicum* extract enhances adipogenesis through PPAR*γ* activation. Hypoadiponectinemia appears to play an important causal role in insulin resistance, type 2 DM and metabolic syndrome [40, 41]. We could not exclude the possibility that butanol fraction affects mRNA stability of those genes, and further studies may require elucidating the molecular mechanism by which butanol fraction affects gene induction during adipogenesis. Since LPL (a PPAR*γ* target gene) did not increase in the presence of butanol fraction, it is possible that butanol fraction may affect gene induction in a PPAR*γ*-independent manner. The data also showed that butanol fraction increased the activity of PPAR*α*; however, further work to investigate expression of the PPAR*α* target gene is required to claim that the extract of *A. indicum* acts as a PPAR*α*/*γ* dual agonist. Indeed, PPAR*α* agonists, such as fibrates, have been used to treat hypertriglyceridemia and reduce cardiovascular risk [42]. Dual PPAR*α*/*γ* agonists hold promise to be able to combine the properties of TZDs and fibrates, as well as improve the management of type 2 diabetes and provide an effective therapeutic option for treating the multifactorial components of cardiovascular disease, metabolic syndrome [43], and even cancer.

Another important finding of this work is that the extract of *A. indicum* possessed considerable insulin-like properties, as evidenced by enhancement of glucose uptake in the diaphragm, which represents muscle cells that are the major site of insulin-stimulated glucose disposal [44]. In the follow-up studies, we examined potential mechanisms underlying the improvement in glucose utilization by the extract. The defective glucose transport system may play an important role in the pathogenesis of peripheral insulin resistance, and glucose uptake in target tissues is a critical step in maintaining glucose homeostasis and in clearing the postprandial glucose load [45]. Thus, we examined the possibility that the extract may stimulate glucose transport through a mechanism similar to insulin signaling in C2C12 myotubes and 3T3-L1 adipocytes. Previous literature has documented that the phosphatidylinositol-3-kinase (PI3K) pathway plays an important role in the insulin signaling cascade leading to glucose transport translocation [44]. However, the results showed that under basal or insulin-stimulated conditions, all of the extract treatments (crude extract, water and butanol fraction) had no effect on Akt serine phosphorylation in either cell type (data not shown). Another pathway that has been implicated in glucose transport, not by translocation of GLUT, but by activation of GLUT, is the p38 pathway [46], which is also interesting for further study. This pathway has been reported to be involved in the regulation of GLUT 1 expression [47]. Subsequently, we examined the effect of the extract on transcription activation of GLUT1. As demonstrated in this study, extract of *A. indicum* activated transcription of GLUT1 in a dose-response manner, suggesting involvement of GLUT1 in increasing glucose uptake by the extract. This result will require confirmation such as measuring extracellular signal regulated kinases (ERKs), which have been known to be responsible for the expression of GLUT1 [48].

**Figure 7:**
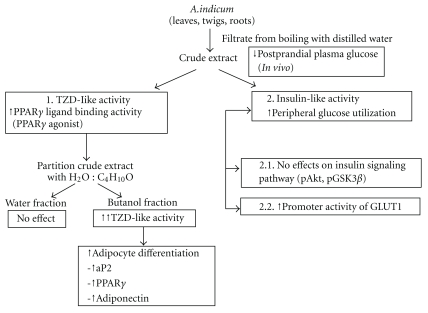
Summary of antidiabetic effects against insulin resistance of *A. indicum* Sweet.

We have reported that the phytochemical analysis of the whole plant extract revealed the presence of alkaloids, flavonoids, and tannins [11]. Results from other researchers have shown that this plant also contains saponins and glycosides [10]. Most plants with antidiabetic properties have been found to contain metabolites such as glycosides, alkaloids and flavonoids [9]. Indeed, seven compounds were isolated from *A. indicum* extract and six of them identified as *β*-sitosterol, oleanolic acid, (24R)-*α*-stigmastane-3,6-dione, daucosterol, 2,6-dimethoxy-1,4-benzoquinone, and vanillic acid [49]. Oleanolic acid has been reported to have hypoglycemic activity by acting as an inhibitor of glycogen phosphorylase, leading to the inhibition of hepatic glycogenolysis [50]. The alkaloid berberine has also been reported to activate GLUT1-mediated glucose uptake [48]. Thus, these results provide evidence that these compounds may lead to affect the antidiabetic activity of *A. indicum*. The findings of antidiabetic properties in this study are summarized in Figure 7, and support the ethnobotanical use of *A. indicum* in the context of human type 2 DM; however, the molecules of action responsible for *A. indicum*'s effect remain to be identified.

## Funding

The Center of Excellence in Livestock Diseases and Human Health at the University of Tennessee and a National Institutes of Health grant (R21CA109423). Financial support was provided by the Royal Golden Jubilee PhD Program (PHD/0245/2545) and the Office of the Higher Education Commission, Thailand (to C.K.).

## Figures and Tables

**Table 1:** Primer sequences used for RT-PCR.

Gene	Sense	Sequence
Adiponectin	Forward	5′-TGC ACA GCT CCG TGT ACT TC-3′
	Reverse	5′-CAC CTG CAC AGA GTC GTC AT-3′

aP2	Forward	5′-AAG AAG TGG GAG TGG GCT TT-3′
	Reverse	5′-CTT GTG GAA GTC ACG CCT TT-3′

LPL	Forward	5′-GGA TCC GTG GCC GCA GCA GAC GCA GGA AGA-3′
	Reverse	5′-GAA TTC CAT CCA GTT GAT GAA TCT GGC CAC-3′

PPAR*γ*	Forward	5′-GGT GAA ACT CTG GGA GAT TC-3′
	Reverse	5′-CAA CCA TTG GGT CAG CTC TT-3′

GAPDH	Forward	5′-CAG GAG CGA GAC CCC ACT AAC AT-3′
	Reverse	5′-GTC AGA TCC ACG ACG GAC ACA TT-3′
